# Is a single dose of meningococcal serogroup C conjugate vaccine sufficient for protection? experience from the Netherlands

**DOI:** 10.1186/1471-2334-12-35

**Published:** 2012-02-08

**Authors:** Patricia Kaaijk, Arie van der Ende, Guy Berbers, Germie PJM van den Dobbelsteen, Nynke Y Rots

**Affiliations:** 1 National Institute for Public Health and the Environment (RIVM), Centre for infectious Diseases Control Netherlands, Section Vaccinology, Bilthoven, the Netherlands; 2 Academic Medical Centre (AMC), Department of medical Microbiology and the Netherlands Reference Laboratory for Bacterial Meningitis, Amsterdam, the Netherlands; 3 National Institute for Public Health and the Environment (RIVM), Centre for infectious Diseases Control Netherlands, Laboratorium for Infectious Diseases and Screening, Bilthoven, the Netherlands; 4 National Institute for Public Health and the Environment (RIVM), Centre for infectious Diseases Control Netherlands, Section Vaccinology, Building A9, Mailbox 41, PO Box 1, 3720, BA Bilthoven, The Netherlands

## Abstract

**Background:**

The first meningococcal serogroup C (MenC) conjugate vaccine was licensed in 1999 and introduced in the United Kingdom. Countries that have implemented the MenC vaccine since then in their national immunisation programmes use different schedules. Nevertheless, all involved countries seem to experience substantial declines in the incidence of MenC disease.

**Discussion:**

Since 2001, the MenC conjugate vaccine has been implemented in the Netherlands by offering a single dose to all children aged 14 months. Prior to the introduction of the vaccine into the national immunisation programme, a catch-up vaccination campaign was initiated in which a single dose of the MenC conjugate vaccine was offered to all children aged from 14 months up to and including 18 years. Since then, there has been no report of any case of MenC disease among immunocompetent vaccinees. Administration of a single dose of MenC conjugate vaccine after infancy could be beneficial considering the already complex immunisation schedules with large numbers of vaccinations in the first year of life. The present paper deals with the advantages and critical aspects of a single dose of the MenC conjugate vaccine.

**Summary:**

A single dose of MenC conjugate vaccine at the age of 14 months in combination with a catch up vaccine campaign appeared to be a successful strategy to prevent MenC disease in the Netherlands, thereby confirming that a single dose of the vaccine could sufficiently protect against disease. Nevertheless, this approach can only be justified in countries with a relatively low incidence of serogroup C meningococcal disease in the first year of life. Furthermore, a good surveillance programme is recommended for timely detection of vaccine breakthroughs and outbreaks among non-vaccinees, since long-term protection after a single dose in the second year of life cannot currently be guaranteed.

## Background

*Neisseria meningitidis *is a major cause of invasive bacterial infections worldwide [1]. The highest incidence rate of meningococcal serogroup C (MenC) disease is in children aged 2 years or younger and in the age group of 14 to 20 years [2]. Efforts to control infections with *N. meningitidis *have been aimed at the development of effective meningococcal vaccines and subsequent implementation in appropriate vaccination schedules. In 1999, an efficacious conjugate vaccine against MenC disease, consisting of serogroup C capsule polysaccharide conjugated to tetanus toxoid as carrier protein, has been licensed in Europe and has been used widely ever since. However, in the absence of randomised controlled trials on efficacy that support a specific vaccination schedule, each country has its own scheme with respect to the MenC conjugate vaccine (Table 1) [3]. In general, regardless of the vaccination schedule that has been followed, all countries that implemented the MenC conjugate vaccine in their programme experienced substantial declines in the incidence of MenC disease [4]. At present, not all European countries have implemented the MenC conjugate vaccine in their national immunisation programmes, such as Scandinavia, and most Eastern European countries. For the countries that have included MenC vaccine in their programme, the most commonly used vaccination schedules are 2 doses in first year of life and a booster in the second year of life or 1 dose in the second year of life with possibly an additional booster later in life. The present paper deals with the advantages and critical aspects of a single dose of the MenC conjugate vaccine in the second year of life with the experience of the Netherlands as example.

**Table 1:** Current vaccination schedules with the MenC conjugate vaccine in European countries

Country	Number of doses	Current MenC vaccination schedule	Source
Andorra	3	2, 4, and 18 months	WHO^a^
Austria	2	13 months (MenC) and 11-13 years (MenACWY)	The Federal Ministry of Health, Austria^b^
Belgium	1	15 months	WHO
Cyprus	1	12-13 months	WHO
France	1	12-24 months	Ministry of labour, employment and health, France^c^
Germany	1	11-23 months	WHO
Greece	3	2, 4, and 15-18 months	WHO
Iceland	2	6 and 8 months	WHO
Ireland	3	4, 6, and 13 months	WHO
Italy	3	3, 5, and 11-12 months and risk groups	WHO
Luxembourg	1	≥ 13 months	WHO
Monaco	1	2 years	WHO
Netherlands	1	14 months	WHO
Portugal	3	3, 5, 15 months	WHO
Slovenia	-	risk groups	WHO
Spain	3	2, 4-6, 12-18 months	Vaccine Advisory Committee of the Spanish Association of Paediatrics^d^
Switzerland	2	12-15 months and 11-15 years	WHO
United Kingdom	3	3, 4, 12-13 months	Department of Health, United Kingdom^e^

^a ^http://apps.who.int/immunization_monitoring/en/globalsummary/scheduleselect.cfm

^b^http://www.bmg.gv.at/cms/home/attachments/1/4/0/CH1100/CMS1038913010412/impfplan_2011.pdf

^c^http://www.sante.gouv.fr/IMG/pdf/Tableau_des_vaccinations_recommandees_chez_les_enfants_et_adolescents.pdf

^d^http://vacunasaep.org/profesionales/calendario-de-vacunaciones-de-la-aep-2011

^e^http://www.dh.gov.uk/prod_consum_dh/groups/dh_digitalassets/documents/digitalasset/dh_122401.pdf

## Discussion

### Implementation of MenC vaccination in the Netherlands

In the Netherlands, the increase in MenC disease in 2000-2002 was the reason to implement the MenC conjugate vaccine into the Dutch immunisation programme (Table 2). In 2000, 105 patients (an annual incidence rate of 0.7 per 100.000 inhabitants of the Netherlands) with a culture-proven serogroup C meningococcal infection were reported and in 2001 this number increased to 276 patients (an annual incidence rate of 1.7 per 100.000 inhabitants of the Netherlands). In the Spring of 2002, just prior to the implementation of the MenC vaccine into the immunisation programme, a vaccination campaign was initiated including all children aged from 14 months up to and including 18 years was started. Subsequently, the MenC vaccine, i.e. a conjugated MenC vaccine with tetanus toxoid as carrier protein, was introduced for children at the age of 14 months into the immunisation programme of the Netherlands from September 2002 onwards.

**Table 2:** MenC disease cases^a ^in the Netherlands per year, age group, and among vaccinees

Year	Total	**Incidence rate**^b^				Age groups					
			**0-11 mths**	**1-4 yrs**	**5-9 yrs**	**10-14 yrs**	**15-19 yrs**	**20-24 yrs**	**25-29 yrs**	**≥ 30 yrs**	**among vaccinees**

**2000**	105	0.7	2	22	15	11	18	9	4	24	n.a.
**2001**	276	1.7	23	50	27	39	65	15	9	48	n.a.
**2002**^c^	222	1.4	13	39	30	26	47	17	5	45	0^c^
**2003**^d^	42	0.3	11	6	0	1	0	6	0	18	0
**2004**	17	0.1	1	1	1	0	0	1	2	11	0
**2005**	4	0.03	0	0	0	0	0	0	0	4	0
**2006**	4	0.03	0	1	0	0	0	0	0	3	0
**2007**	9	0.06	2	0	1	0	1	1	0	4	0
**2008**	11	0.07	2	0	0	0	0	0	1	8	0
**2009**	10	0.06	1	1	0	0	1	0	2	5	0 (1)^e^
**2010**	6	0.04	2	0	0	0	2	0	0	2	0 (1)^e^

Surveillance data 2000-2009 are obtained from annual reports "Bacterial Meningitis in the Netherlands" published by "Netherlands Reference Laboratory for Bacterial Meningitis" [5]. Data from 2010 are not published yet

n.a.; not applicable

^a ^MenC disease cases are defined as culture and/or PCR-proven presence of MenC bacteria in cerebrospinal fluid and/or blood

^b ^Incidence rate is defined as the annual incidence rate of MenC disease per 100.000 inhabitants of the Netherlands

^c ^In the Netherlands, MenC conjugate vaccine was introduced in Spring 2002

^d ^The case fatality rate (CFR) calculated from 58 patients with MenC disease cases occurring between January 2003-May 2005 was 5.2% [6]

^e ^One case of MenC disease occurred in a vaccinated 16 years old female (2009), and one case in a vaccinated 19 years old male (2010). Both persons had an immune disorder

During this vaccination campaign approximately 3.5 million vaccinations were given, which together with the vaccinations given prior to the campaign resulted in a vaccination rate of 94% of the target population. At present, the vaccination rate among children receiving a single MenC conjugate vaccine dose at the age of 14 months according to the Dutch immunisation programme has been reported to be as high as ≥ 95% (birth cohort 2007) [7].

### Outcome of MenC vaccine implementation in the Netherlands

In the Netherlands, surveillance of MenC disease is performed by a central laboratory that receives nation-wide meningococcal isolates from more than 90% of all cases with invasive meningococcal disease. Strikingly, since the introduction of MenC conjugate vaccine in the Netherlands there has been no report of any case of MenC disease among immunocompetent vaccinees (Table 2). In addition, the MenC disease incidence among non-vaccinees showed a clear reduction due to herd immunity. From 2004 onwards, a maximum of 2 cases of MenC disease per year occurred at the age < 1 year (all infant cases occurred at the age ≥ 6 months, < 1 year). Apparently, the routine childhood vaccination and the initial catch-up campaign resulted in a reduction of circulating serogroup C meningococci which in turn has led to a decreased transmission. It is important to note that replacement by meningococci with other serogroups, i.e. serogroup A, B, Y, W135, was not observed. On the contrary, the incidence of serogroup B meningococcal disease decreased from 1998 onwards and continued to decline since the introduction of the MenC conjugate vaccine in 2002 [5]. However, this is considered to be based on coincidence, since the MenC vaccine does not induce cross-reactive immunity to meningococcal serogroup B bacteria. This is confirmed by a study in the United Kingdom, whereby the prevalence of carriage of meningococci associated with serogroup B disease was unaffected by the introduction of the MenC conjugate vaccine [8]. As expected, in this latter study a decline in prevalence of carriage of serogroup C meningococci was reported from 1999, the year of introduction of MenC conjugate vaccine in UK. This finding was consistent with the observed herd immunity in the non-vaccinated teenage population in the UK [8].

### Duration of immune response afforded by MenC vaccine implementation in the Netherlands

Recently, MenC-specific immunity before and after implementation of the MenC conjugate vaccine in 2002 in the Netherlands has been analysed using two large cross-sectional serum banks each obtained from a nation-wide Dutch population [9]. These serum banks contain samples from participants 0-79 years of age that were collected in two cross-sectional serosurveillance studies performed in 1995/6 and 2006/7 [10,11]. From these serum banks, polysaccharide specific IgG concentrations against serogroup C meningococci and functional antibody titers obtained with the SBA have been determined and described earlier [9]. Here we show the SBA titres from respectively 735 and 1220 serum samples before and after the introduction of the MenC conjugate vaccine (Table 3). These data illustrate that, more than 4 years after vaccination (serum bank 2006/7), the MenC-specific SBA titres were still remarkably high (SBA geomean titre (GMT) ≥ 8) in individuals who were vaccinated at the age of 5 year or older during the vaccination campaign (aged 9/10 years in 2006/7) (Table 3). A single MenC conjugate vaccine administration above 5 years of age seems to induce persistent protective SBA antibody levels, gradually increasing with age of vaccination. In contrast, MenC-specific SBA levels in serum samples from individuals that have been vaccinated against MenC disease at the age of 14 months, i.e. according to the Dutch routine immunisation programme, showed a rapid decline within a few years after vaccination. Apparently, high functional antibody levels against serogroup C meningococci persist longer in persons who were vaccinated at an age of 5 year or older [9].

**Table 3:** Serum bactericidal antibody (SBA) titres from serum samples obtained before and after the introduction of the MenC conjugate vaccine [9]

Age	Pre-introduction MenC vaccine (1995/6)			Post-introduction MenC vaccine (2006/7)		
	
	No. samples	SBA-GMT (95% CI)	% samples SBA ≥ 8	No. samples	SBA-GMT (95% CI)	% samples SBA ≥ 8
0-7 mths	3	2 (NA)	0	59	2 (n.a.)	0
8-14 mths	13	2.1 (1.9-2.4)	0	62	2.4 (1.9-3.0)	3.2
15-24 mths	18	2.0 (NA)	0	27	131.3 (64.5-267.5)	92.6
2 yrs	24	2.0 (NA)	0	42	13.1 (8.3-20.8)	61.9
3-4 yrs	42	2.1 (1.9-2.4)	2.6	106	5.4 (3.8-7.7)	30.2
5-6 yrs	34	2.8 (1.6-4.7)	6	49	5.2 (3.4-7.8)	28.6
7-8 yrs	43	3.4 (2.2-5.2)	11.2	56	5.1 (3.7-7.1)	33.9
9-10 yrs^a^	27	3.0 (1.3-6.9)	11.3	73	9.4 (5.8-15.2)	45.2
11-12 yrs	31	2.5 (1.4-4.5)	8.4	72	20.0 (12.4-32.2)	61.1
13-14 yrs	34	5.6 (2.1-14.8)	23.5	65	23.5 (15.9-34.6)	69.2
15-16 yrs	38	3.3 (2.1-5.1)	16.3	55	57.9 (36.7-91.1)	81.8
17-18 yrs	25	3.6 (1.8-7.1)	11.7	43	89.8 (58.4-138.0)	86
19-21 yrs	21	2.6 (1.9-3.5)	5.9	88	159.6 (109.1-233.4)	95.5
22-25 yrs	38	6.2 (3.1-12.4)	26.1	104	4.2 (9.1-22.6)	49
26-30 yrs	56	4.6 (2.1-10.0)	24.5	69	4.2 (3.1-5.9)	26.1
31-39 yrs	66	4.7 (3.8-5.8)	22.5	58	3.6 (2.1-6.0)	19
40-49 yrs	65	5.3 (4.0-6.9)	26	49	4.1 (2.5-6.7)	20.4
50-59 yrs	59	4.5 (3.9-5.2)	19.2	52	4.1 (2.5-6.6)	21.2
60-69 yrs	51	5.9 (3.0-11.5)	29.5	54	3.2 (2.7-3.7)	18.5
70-79 yrs	48	3.6 (2.1-6.1)	17.1	37	3.6 (2.5-5.4)	21.6
Total	736	4.3 (3.3-5.5)	19.7	1220	10.2 (8.9-11.7)	43.0

n.a.; not applicable

95% CI; 95% confidence interval

^a ^Individuals who were vaccinated at the age of 5 year during the vaccination campaign are aged 9-10 years in 2006/7

These findings are consistent with results from a study in the United Kingdom, showing that 5 years after vaccination children vaccinated at an age of 10 years or older exhibited higher functional (SBA) antibody levels against serogroup C meningococci compared to younger children [12]. Although, at present SBA is the best correlate of protection for evaluating the immune response to meningococcal serogroup C vaccines, for long-term protection SBA levels alone may not be ideal [13]. A better predictor for long-term protection may be a combination of SBA levels with the number of MenC-specific memory B cells [14]. At present, failures of long-term protection in children immunised with a single dose of MenC conjugate vaccine at the second year of life have not been documented [3]. The question that thus remains is whether the existing antibody response and/or B cell memory response in the individuals vaccinated with a single dose at the age of 14 months will be strong enough to prevent disease in case of exposure to serogroup C meningococci at later ages. High SBA levels may be necessary, because meningococci have the potential of rapid multiplication leading to fast progression of MenC disease [15,16]. This is probably why some countries (i.e. Austria and Switzerland) have implemented a booster dose at adolescent age in addition to the vaccine dose in the second year of life (Table 1). So far, no breakthroughs of MenC disease have occurred in immunocompetent vaccinated individuals in the Netherlands. This is most probably due to diminished circulation of serogroup C meningococci as a consequence of herd immunity induced by vaccination. On the other hand, it might be that all vaccinated individuals are protected, despite the low SBA levels in those who have been vaccinated at younger ages.

### Implementation of the MenC vaccine in other countries

#### MenC vaccination in first year of life

In 1999, the MenC conjugate vaccine was introduced nation-wide in the United Kingdom as first country in Europe using three different MenC conjugate vaccines. The vaccination scheme of the UK was three doses at infancy, at the age of 2, 3 and 4 months. In Ireland and Spain, similar MenC vaccination schedules were implemented with three doses in the first year of life. In 2006, the MenC vaccination schedule of the UK was altered into the three dose schedule at 3, 4 and 12-13 months of age. This decision was based upon the evidence-based assumption that a booster with MenC conjugate vaccine in the second year of life is needed to maintain protection against MenC disease after infancy and that two doses for infants is minimally needed for a good priming response [17,18]. At present, the vaccination schemes in Ireland and Spain have also been adapted accordingly with two doses of the MenC conjugate vaccine in the first year of life and a third booster dose in the second year of life. A comparable vaccination scheme is implemented in Andorra, Greece and Italy (Table 1). Results from the different immunisation schedules that have been used in Canada revealed that multiple doses in early infancy provides little additional benefit over programmes starting with 1 dose at the age of 12 months [19].

#### MenC vaccination in second year of life

At present, apart from the Netherlands, several other countries, such as Belgium, Cyprus, France, Germany, Luxembourg, and Monaco, have implemented a vaccination scheme with a single dose in the second year of life (Table 1). However, this approach can only be justified in countries with a relatively low incidence of serogroup C meningococcal disease in the first year of life, which was the case in the Netherlands prior to introduction of the MenC conjugate vaccine (< 10% of MenC disease cases occurred in infancy; Table 2). Recently, Austria and Switzerland have introduced an additional booster dose in teenagers besides the primary single dose in the second year of life. This vaccination schedule anticipates the observed waning immunity found after 1 dose in the second year of life.

The ideal immunisation schedule should aim at a maximal level of effectiveness with a minimum number of doses needed. A single vaccination in the second year of life might be sufficient for adequate protection, whereas a minimum of 2 doses seems necessary when starting in early infancy [17,18]. The immune system of younger infants is not fully developed. For example, a conjugated Hib vaccine induced a significantly lower antibody response at 2-3 months of age when compared to 6 months or later [20]. For this reason, administration of MenC conjugate vaccine after infancy, such as has been implemented in the Netherlands, could be beneficial. In addition, most national immunisation programmes includes already a large number of vaccinations in the first year of life. This makes the introduction of newly available vaccines at infancy, such as against rotavirus, increasingly difficult, while there are still several new vaccines under development for this young age group, such as vaccines against serogroup B meningococci and respiratory syncytial virus.

## Summary

Despite the fact that different schedules have been used for implementing the MenC conjugate vaccine, all involved countries seem to experience substantial declines in the incidence of MenC disease [4]. In the Netherlands, there has been no report of any case of MenC disease among immunocompetent vaccinees, since the introduction of a single dose of the MenC conjugate vaccine in the second year of life. Although no data on nasopharyngeal carriage rates before and after immunisation are available, it is likely that the combination of this introduction together with the vaccine campaign, in which a single vaccine dose was offered to all children aged from 14 months up to and including 18 years, has led to a major reduction of circulating serogroup C meningococci. The herd immunity that was established has also led to a significant reduction of serogroup C meningococcal disease among non-vaccinees. Nevertheless, it had been shown that individuals vaccinated at the age of 14 months with a single dose of the MenC conjugate vaccine showed a rapid decline in (functional) antibody levels against serogroup C meningococci within a few years after vaccination. For this reason, when applying this vaccination scheme, i.e. a single dose in the second year of life without an addition booster dose, it is important to have a solid surveillance programme, in which the number of patients with serogroup C meningococcal disease is carefully being monitored in order to determine long-term vaccine protection. This will allow timely detection of vaccine breakthroughs and outbreaks among non-vaccinees, making a timely and appropriate intervention possible, such as deciding to administer a booster vaccination when needed to guarantee protection during periods of susceptibility in the second decade of life.

## Competing interests

The authors declare that they have no competing interests.

## Authors' contributions

PK drafted the outline and the text of the manuscript. AE was responsible for the surveillance data of MenC disease in the Netherlands, GB for the sero-epidemiological data of MenC, GD as expert on meningococcal disease provided intellectual content that added to the manuscript and NR highlighted the need for this debate. All authors were actively involved in reviewing the content and editing the text of the manuscript. All authors read and approved the final version of the manuscript.

## Pre-publication history

The pre-publication history for this paper can be accessed here:

http://www.biomedcentral.com/1471-2334/12/35/prepub
